# Effect of Cyclic Stretch on Tissue Maturation in Myoblast-Laden Hydrogel Fibers

**DOI:** 10.3390/mi10060399

**Published:** 2019-06-15

**Authors:** Shinako Bansai, Takashi Morikura, Hiroaki Onoe, Shogo Miyata

**Affiliations:** 1 Graduate School of Science and Technology, Keio University, 3-14-1 Hiyoshi, Yokohama 223-8522, Japan; shinako0220@keio.jp (S.B.); dnngu-1elife@keio.jp (T.M.); 2 Department of Mechanical Engineering, Faculty of Science and Technology, Keio University, 3-14-1 Hiyoshi, Yokohama 223-8522, Japan; onoe@mech.keio.ac.jp

**Keywords:** myoblast, skeletal muscle, core-shell hydrogel fiber, cyclic stretch, engineered muscle

## Abstract

Engineering of the skeletal muscles has attracted attention for the restoration of damaged muscles from myopathy, injury, and extraction of malignant tumors. Reconstructing a three-dimensional muscle using living cells could be a promising approach. However, the regenerated tissue exhibits a weak construction force due to the insufficient tissue maturation. The purpose of this study is to establish the reconstruction system for the skeletal muscle. We used a cell-laden core-shell hydrogel microfiber as a three-dimensional culture to control the cellular orientation. Moreover, to mature the muscle tissue in the microfiber, we also developed a custom-made culture device for imposing cyclic stretch stimulation using a motorized stage and the fiber-grab system. As a result, the directions of the myotubes were oriented and the mature myotubes could be formed by cyclic stretch stimulation.

## 1. Introduction

Muscle tissue consists of an ordered muscle fiber array, which is tightly bundled, long, and cylindrical multinucleated myotube cells. Muscles play an important role in daily human activities including metabolic regulation of internal organs. Myopathy, injury, and extraction of malignant tumors are some of the common issues to restore muscle tissue. Therefore, the tissue engineering approach for muscle regeneration is beneficial. There are several approaches to reconstruct the three-dimensional muscle tissue: Cell-seeded collagen gel [1], cell-based sheets [2], cell aggregates [3], etc. In addition, aligned electrospun nanofibers are also used for scaffold materials, to promote cellular alignment [4,5]. These approaches also have the potential for in vitro drug screening and disease modeling [6,7,8,9]. Muscle tissue can be regenerated by these approaches. However, the maturation of the engineered tissue is still far from the “native” muscle [10].

In this study, we control the orientation of myoblast-like cells and reconstruct the maturated muscle tissues by mechanical stimuli. The effect of mechanical stimulation on cell homeostasis and development, which are critical factors in tissue maintenance, repair, and regeneration, has drawn a lot of attention. As mechanical stimuli to promote tissue regeneration, the stimuli mimicking in vivo physiological condition was imposed on living cells: Shear stress or stretch for blood vessel remodeling [11,12], stretch for the bone [13] and ligament [14] remodeling, and stretch or electric stimuli [15,16] for muscle remodeling. For myogenesis, the mechanical and chemical stimulations promote the myogenesis of myoblasts or myoblast-like cells to become multinucleated myotube cells [17]. It has been reported that mechanical stretch can affect the remodeling of the cytoskeleton in myocytes [18,19]. Considering the results of previous studies, mechanical stretch was used as the stimuli in this study. Nguyen et al. already developed the cell culture device to impose cyclic stimuli on a cell-seeded sheet-shaped scaffold and reported the effect of mechanical stimuli on fibrous tissue reconstruction [20]. However, their research was performed regarding the fibroblast culture on the sheet-shaped scaffold, which was not suitable to simulate muscle tissue. Skeletal muscle has the possibility to restore itself after minor injury. However, promotion of myogenesis by mechanical stimuli also benefits cardiac muscle tissue engineering and has drawn a lot of attention in severe cardiac disease cases. 

To mimic the “native” muscle structure, we focused on cell fiber technology [21]. This technology encapsulates living cells into the core region of a hydrogel core-shell microfiber, allowing the cells to grow, migrate, promote cell-cell interaction, and form a fiber-shaped tissue called “cell fiber”. Using this cell fiber technology based on the hydrogel tube structure, gases (O_2_ and CO_2_) and nutrients are allowed to penetrate into the core region containing cells [22], leading to an efficient cell expansion with high viability. 

Here, we develop a custom-made culture device for “cell fiber” to impose mechanical stretch cyclically on the cell fiber using a motorized stage and the fiber-grab system. In addition, we also evaluate the effect of the cyclic stretch on in vitro skeletal muscle regeneration. 

## 2. Materials and Methods 

### 2.1. Cells

Mature murine myogenic cell line C2C12 cells were purchased from Riken Cell Bank (Tsukuba, Japan). The culture medium was Dulbecco’s modified essential medium (DMEM, Sigma, St. Louis, MO, USA) containing 10% fetal bovine serum (FBS), and 1% antibiotic/antimycotic solution (A/A, Thermo Fisher Scientific, Waltham, MA, USA). The cells were maintained in a 5% CO_2_ atmosphere at 37 °C in a CO_2_ incubator and used for experiments before they reached 5 passages. 

### 2.2. Formation of Core-Shell Hydrogel Microfibers

**Figure 1:**
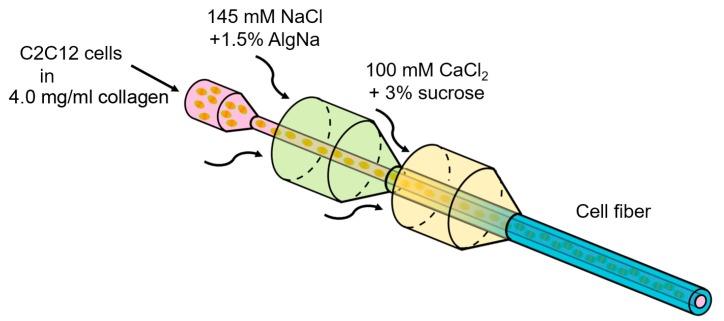
Schematic for fabrication of core-shell hydrogel microfibers. The C2C12 cell-laden core-shell hydrogel microfiber was formed by the double co-axial laminar flow.

According to previous studies, C2C12 cells were cultured in collagen gel [23,24]. To encapsulate C2C12 cells suspended in the collagen gel in the core region of alginate fibers, the double-coaxial laminar-flow microfluidic device was fabricated by assembling pulled glass capillary tubes, rectangular glass tubes, and custom-made three-way connectors, as previously described (Figure 1) [16]. Three solutions were required for core-shell hydrogel microfiber formation: (1) core stream: A solution of C2C12 cells suspended in 4.0 mg/mL neutralized type I collagen (AteloCell^®^, IC-50, KOKEN, Tokyo, Japan) at 1.8 × 10^8^ cells/mL, (2) shell stream: A solution of 1.5 wt % sodium alginate (80–120 cP, Wako Pure Chemical Industries, Osaka, Japan), and (3) sheath stream: A solution of 100 mM calcium chloride (CaCl_2_, Kanto Chemicals, Tokyo, Japan) with 3% w/w sucrose (Nacalai Tesque, Kyoto, Japan). The flow rates of the core, shell, and sheath streams were 25 μL/min, 120 μL/min, and 3.6 mL/min, respectively. The fabricated fibers were finally cultured in the culture medium for 24 h to induce the collagen gelation and for cell adhesion.

**Figure 2:**
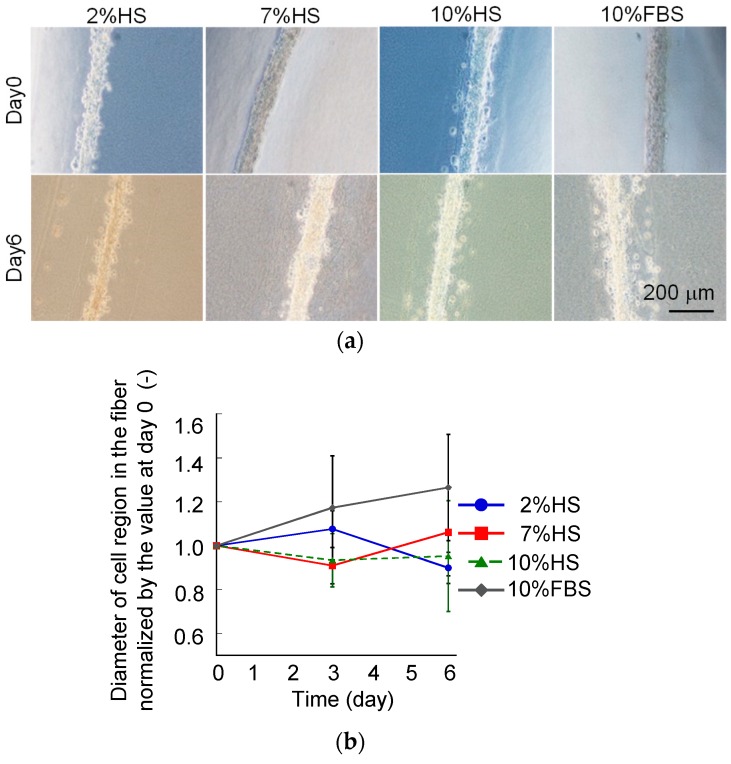
Cell-laden core-shell hydrogel microfiber culture under 2%, 7%, and 10% horse serum (HS) and 10% fetal bovine serum (FBS) conditions. (**a**) Phase-contrast images and (**b**) the change in the diameter of cell-laden core along with culture time. Scale bar: 200 μm.

The differentiation protocol for C2C12 cells were already standardized. However, that for the three-dimensional culture of C2C12 cells were not fully established. To validate the culture medium for three-dimensional culture of C2C12 cells, three types of medium, DMEM with 2% horse serum (HS) [25], 10% HS, and 10% FBS, were tested for preliminary study. As a result, the cells in fibers cultured in DMEM with 2% and 10% HS tended to decrease whereas the cells cultured in DMEM with 10% FBS tended to increase in a 6-day culture (Figure 2). Based on this data, DMEM with 10% FBS was determined as the culture medium for C2C12-cell fibers. 

### 2.3. Three-Dimensional Cell Culture with Cyclic Stretch

**Figure 3:**
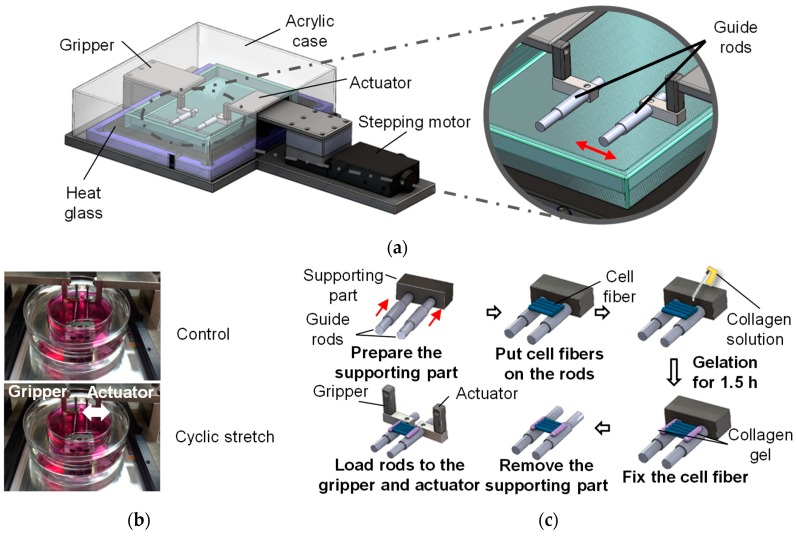
Custom-made cell culture device for “cell fiber” to impose the cyclic stretch. (**a**) Schematic of the culture device, (**b**) photograph of the gripper for hydrogel fibers, and (**c**) procedure to grab the fibers using the collagen gel and two stainless rods.

To impose cyclic stretch on the cell fibers, a custom-made stretching device was developed. The device was composed of a motorized stage and a culture chamber containing two guide rods to hold the cell fiber (Figure 3a). The cell fibers were wrapped around two parallel rods to stretch the cell fibers and the distance of the rods was changed cyclically using a computer controlled motorized stage (Figure 3b). Briefly, the guide rods were set to be parallel (10 mm apart) using a supporting block and the fibers were wrapped around the rods. After the fiber wrapping, 4.0 mg/mL type I collagen solution was dropped on the connecting part of the fibers and the guide rods to ensure the adhesion. Following collagen gelation, the rods were removed and connected to the custom-made stretching device (Figure 3c). The chamber was then filled with 5 mL culture medium to immerse the fibers in the medium. The stretching device was set in a CO_2_ incubator to culture the fibers in a 5% CO_2_ atmosphere at 37 °C. After 2-day static culture, the cell fibers were subjected to 3% tensile strain at 1 Hz for 4 h/day for 2 days. The tensile strain and frequency were decided according to previous studies to avoid the destruction of hydrogel fibers [26]. For control specimens, the cell fibers were cultured under same condition except for the cyclic stretch. 

### 2.4. Microscopy and Image-Based Analysis

To evaluate the myogenesis of C2C12 cells, phase-contrast images were acquired after the 4-day culture (2-day static culture following a 2-day cyclically stretch stimulation). The cells in hydrogel fibers were also stained with calcein-AM to evaluate the morphology of live cells, with rhodamine-phalloidin to evaluate the cytoskeleton. The calcein-AM stains cytoplasm of live cells and the rhodamine phalloidin stains actin filaments. For the calcein-AM staining, the fibers were firstly washed with a serum-free medium two times and incubated with 0.1 mg/mL calcein-AM in DMEM for 30 min. For the rhodamine-phalloidin staining, the cells in the fiber were fixed with 4% paraformaldehyde for 10 min following permeabilization with 0.1% Triton X-100 in phosphate buffered saline (PBS) for 5 min at room temperature. After cell fixation, the cell fibers were incubated with 0.7% rhodamine-phalloidin (PHDR1, Cytoskeleton) for 30 min at 37 °C. After fluorescent staining, the cells were observed by a fluorescent microscope (CKX41, Olympus, Tokyo, Japan) equipped with a CCD camera (DP73, Olympus) and a confocal scanning microscope (FV10i-DOC, Olympus).

To evaluate the myogenesis of C2C12 cells from fluorescent images, the image-based analysis was performed using Image J software (NIH). The fluorescent images were preprocessed using a smooth filter and a sharpen filter with 3 × 3 neighborhood. After the preprocessing, the images were converted to 8-bit grayscale images and binarized using Otsu’s method. Finally, the cell regions in the binary images were fitted to ellipses and the aspect ratio of each ellipse was measured. In this study, cultured C2C12 cells were divided into three groups: (1) undifferentiated cells (aspect ratio < 2.0), (2) immature myotube-like cells (2.0 ≤ aspect ratio < 3.0), and (3) mature myotube-like cells (aspect ratio ≥ 3.0).

## 3. Results and Discussion

### 3.1. Difference in Tissue Remodeling in the Cell Fibers and in the Two-Dimensional Culture

**Figure 4:**
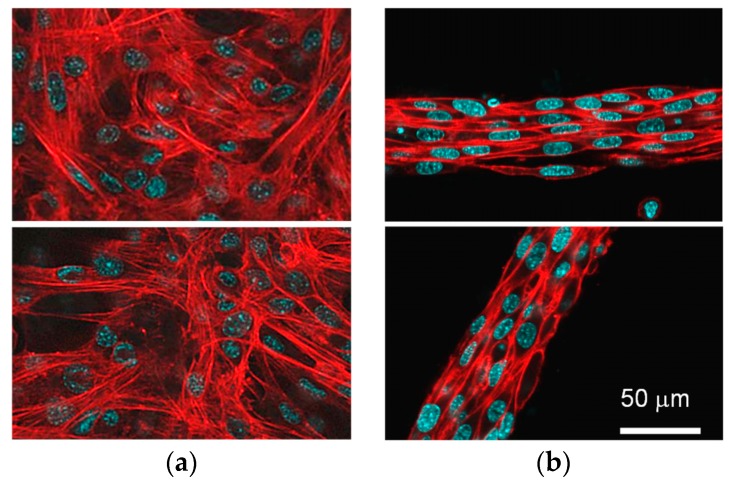
Fluorescent images of rhodamine-phalloidine/DAPI counterstaining to visualize the actin cytoskeleton of the C2C12 cells. (**a**) Monolayer culture and (**b**) three-dimensional culture using a hydrogel microfiber culture of the C2C12 cells. Scale bar: 50 μm.

To evaluate the effect of the three-dimensional culture condition on myogenesis of C2C12 cells, the cytoskeletons of both the monolayer culture and the cell fiber were evaluated (Figure 4). The direction of the cytoskeleton in the monolayer culture was random, whereas the cytoskeleton in the cell fibers aligned to the cylindrical axis of the fiber. It was suggested that the C2C12 cells reorganized their structure of cytoskeleton to align the wall of the gel fiber. Many studies reported that the direction of the cells aligned to the groove of the culture substrate to reorganize the cytoskeleton of the cell [27,28,29,30]. The result of our study was consistent with these studies.

### 3.2. Effect of Cyclic Stretch on Tissue-Reconstruction in the Cell Fibers

**Figure 5:**
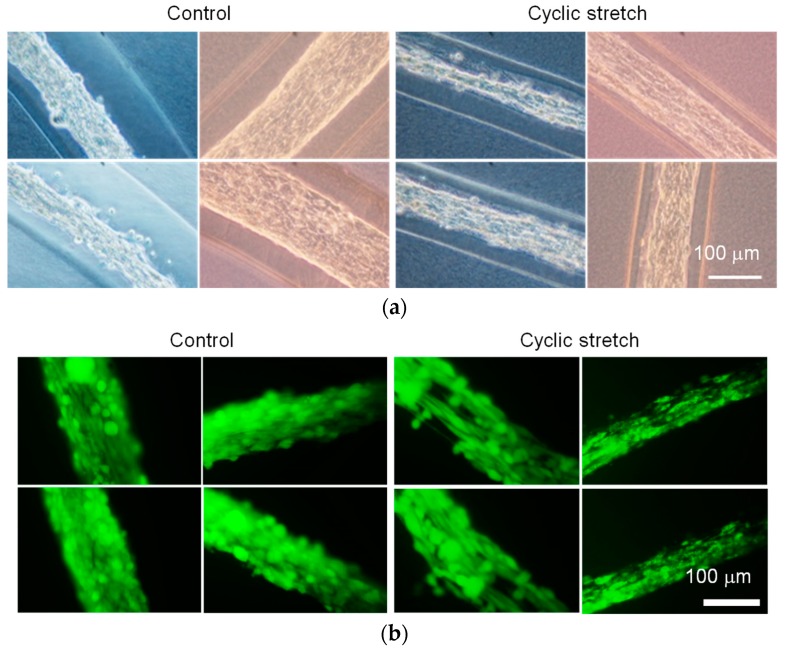
(**a**) Phase-contrast and (**b**) fluorescent images (calcein-AM staining) of C2C12-cell laden hydrogel fibers subjected to cyclic stretch. Scale bar: 100 μm.

**Figure 6:**
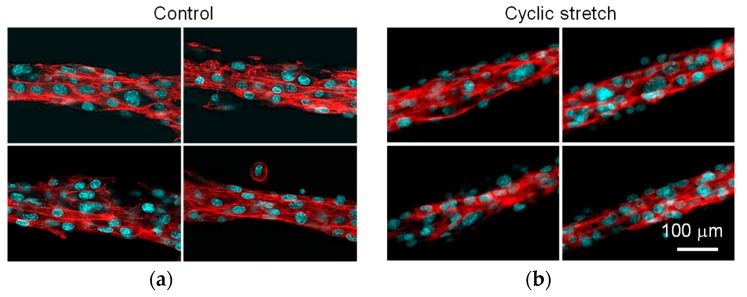
Fluorescent images of rhodamine-phalloidine/DAPI counterstaining to visualize the actin cytoskeleton of C2C12 cells in (**a**) control and (**b**) cyclic stretch groups. Scale bar: 100 μm.

**Figure 7:**
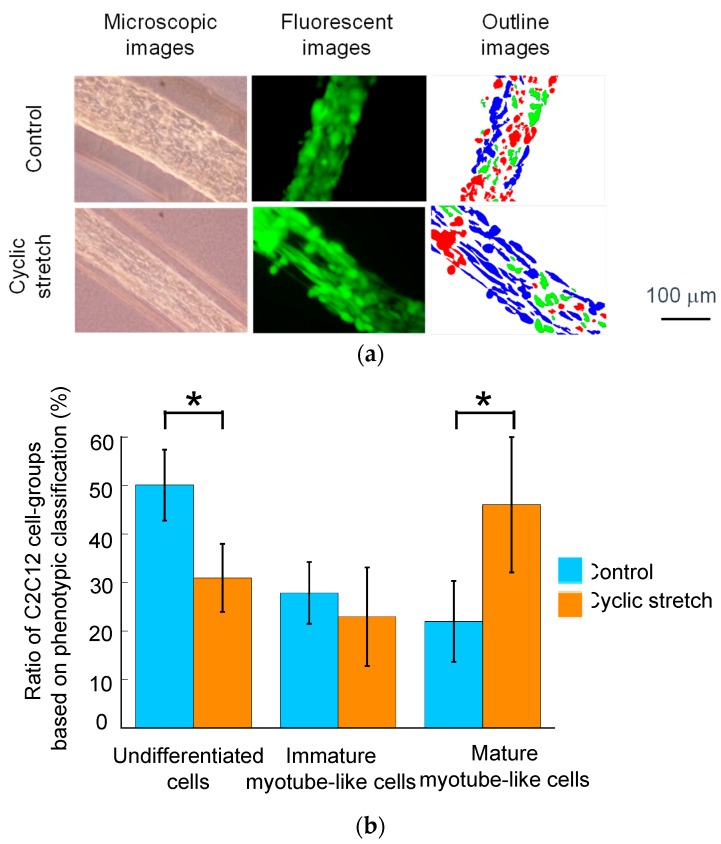
(**a**) Image-based classification of cells (blue: Mature, green: Immature, and red: Undifferentiated) and (**b**) the ratio of three types of C2C12 cells in the cell laden microfibers. A * indicate a significant difference (*p* < 0.05) between control and cyclic stretch groups. Scale bar: 100 μm.

The diameter of the C2C12-cell region in the cell fibers subjected to the cyclic stretch decreased as compared to that in the control group (Figure 5a). Using calcein-AM staining, almost all the cells were positively stained in both the cyclic-stretch and the control group (Figure 5b). This result indicates that the cell viability was maintained in our custom-made cell culture device. The cells in the cyclic-stretch group elongated themselves and aligned to the axis of the fiber. The cells in the control group, on the other hand, were uniformly distributed and did not elongate themselves. As shown in Figure 6, the actin cytoskeleton of the cells in the cyclic-stretch group was concentrated and also aligned to the axis of the cell fiber as compared to the ones in the control group. In this study, we assessed myogenesis of the C2C12 cells based on the aspect ratio of each cell (Figure 7). Cyclic stretch promoted the myogenesis of the C2C12 cells and increased the ratio of the mature myotube-like cells as compared to the ones in the control group. Approximately 70% of the cells were differentiated in the cyclic-stretch group whereas approximately 50% of the cells were differentiated in the control group. Moreover, the ratio of the mature myotube-like cells in the cyclic stretch group was over two times larger than that of the cells in the control group. 

In order to reconstruct the skeletal muscle tissue for the tissue engineering therapy, it is important to culture the cells three-dimensionally with physical stimuli. For in vitro skeletal muscle regeneration, various physical factors are reported to align the cells and progress tissue maturation [15,16,27,28,29,30,31]. Among them, mechanical stress-like tension or electrical stimuli have been reported to affect the cell alignment and maturation in vitro [15,16]. For myoblasts or myoblast-like cells, the stretch could enhance the myosin expression to promote myogenesis [26]. Consistent with these studies, it was considered that the cyclic stretch promoted the myogenesis of the C2C12 cells and the maturation of the muscle fibers in cell-laden hydrogel fibers. Especially, cells in the skeletal muscles, also in addition to the cardiac muscles, are constantly subjected to cyclic mechanical stretch to generate highly differentiated and maturated muscle fibers. Therefore, mechanical stimuli could be an important factor for tissue regeneration of the skeletal and the cardiac muscles.

In addition, for the reconstruction of the three-dimensional muscle tissue, it is important to maintain cell viability in the tissue over the culture time. In this study, the C2C12 cells were contained in the core-shell hydrogel fiber, which is suitable for three-dimensional tissue reconstruction [21]. Moreover, the hydrogel fiber structure could induce exchange of O_2_, CO_2_, and nutrients [32]. Therefore, our skeletal muscle reconstruction system using the cell-laden hydrogel fiber and mechanical stretching stimuli is anticipated to be applicable for in vitro tissue regeneration and clinical applications.

## 4. Conclusions

This study established an in vitro muscle regeneration system to use a cell-laden hydrogel fiber culture and to develop a custom-made culture device to impose the cyclic stretch stimulation on the hydrogel fiber. From the results, it was revealed that the core-shell hydrogel fiber structure could simulate “native” muscle fibrous structure to maintain the cell and muscle fiber alignment. The mechanical stretch could also promote myogenesis and maturation of muscle fibers in the cell-laden hydrogel fibers. In conclusion, our three-dimensional muscle cell culture system with mechanical stimuli could be a promising approach for tissue engineering therapy and its clinical applications.
